# The Half-Yearly Abstract of the Medical Sciences

**Published:** 1848-09

**Authors:** 


*The Half-Yearly Abstract of the Medical Sciences.* By WM. H. RANKING, M. D., etc.; assisted by W. A. GUY, M. D., GEORGE DAY, M. D., HENRY ANCELL, M. D., and W. KIRKES, M. D. No. 7—January to June, 1848. Lindsay & Blakiston. Philadelphia. 1848.

We have not yet received our London copy of No. 7 of Ranking's Abstract, but already we have the re-print of it by Lindsay & Blakiston. From this fact our readers will see that they have a better chance of getting the work promptly from the American than the foreign publishers.

In the number before us, we find the same rich and judicious selection as in preceding numbers, which received our unqualified commendation. The *Reports*, too, which we regard as a valuable feature in the work, are drawn up with great ability. The following are contained in the present number. 1. *Report on the progress of Practical Medicine, Pathology, and Therapeutics*, by the Editor, Dr. Ranking. 2. *On the progress of Surgery*, by H. Ancell, Esq., etc. 3. *On the progress of Midwifery, and Diseases of Women and Children*, by the Editor. 4. *On the progress of Forensic Medicine*, by Dr. Guy. Beside these, there is a supplementary report *On the recent progress of Psycological Medicine*, by Dr. Lockhart Robinson.

The Philadelphia publishers have done justice to the volume, by the neat and careful manner in which it is printed and brought out.

